# Audiological Diagnosis of Valvular and Congenital Heart Diseases in the Era of Artificial Intelligence

**DOI:** 10.31083/j.rcm2406175

**Published:** 2023-06-14

**Authors:** Aikeliyaer Ainiwaer, Kaisaierjiang Kadier, Lian Qin, Rena Rehemuding, Xiang Ma, Yi-Tong Ma

**Affiliations:** ^1^Department of Cardiology, Xinjiang Medical University Affiliated First Hospital, 830011 Urumqi, Xinjiang, China

**Keywords:** artificial intelligence, congenital heart disease, deep learning, diagnosis, valvular heart disease, electronic stethoscope

## Abstract

In recent years, electronic stethoscopes have been combined with artificial intelligence (AI) technology to digitally acquire heart sounds, intelligently identify valvular disease and congenital heart disease, and improve the accuracy of heart disease diagnosis. The research on AI-based intelligent stethoscopy technology mainly focuses on AI algorithms, and the commonly used methods are end-to-end deep learning algorithms and machine learning algorithms based on feature extraction, and the hot spot for future research is to establish a large standardized heart sound database and unify these algorithms for external validation; in addition, different electronic stethoscopes should also be extensively compared so that the algorithms can be compatible with different. In addition, there should be extensive comparison of different electronic stethoscopes so that the algorithms can be compatible with heart sounds collected by different stethoscopes; especially importantly, the deployment of algorithms in the cloud is a major trend in the future development of artificial intelligence. Finally, the research of artificial intelligence based on heart sounds is still in the preliminary stage, although there is great progress in identifying valve disease and congenital heart disease, they are all in the research of algorithm for disease diagnosis, and there is little research on disease severity, remote monitoring, prognosis, etc., which will be a hot spot for future research.

## 1. Introduction

Valvular heart disease (VHD) is a condition in which the valves of the mitral, tricuspid, aortic and pulmonary valves become diseased due to rheumatic fever, mucus degeneration, degenerative changes, congenital malformations, ischemic necrosis, infection or trauma, which affects the normal flow of blood and thus causes abnormal heart function [1]. Approximately 2 million people in China suffer from VHD, and 150,000 new cases of VHD are diagnosed each year [2]. Congenital heart disease (CHD) is defined as a gross structural abnormality of the heart or great vessels [3], common diseases in this category include atrial septal defects (ASDs), patent foramen ovale, ventricular septal defects (VSDs), and patent ductus arteriosus (PDA).

Although imaging tools are the primary methods for diagnosing VHD and CHD, physical examination, which includes cardiac auscultation, is a screening tool for VHD and CHD. Auscultation plays a key role in the diagnosis of VHD and CHD [4, 5, 6]. In the context of analysing heart sound signals, computer-aided detection technology can be a useful and cost-effective tool for acquiring and analysing these signals in a quantitative manner, with the added benefits of speed and efficiency [7].

We performed a narrative literature review, and here, we review the recent progress achieved using machine learning applications with heart sound signals derived from VHD and CHD. We examine the advantages and limitations of using artificial intelligence (AI) techniques in the field of VHD and CHD auscultation and suggest some promising future research directions in this field.

## 2. Overview of Heart Sounds and Heart Murmurs

**Fig. 1.**
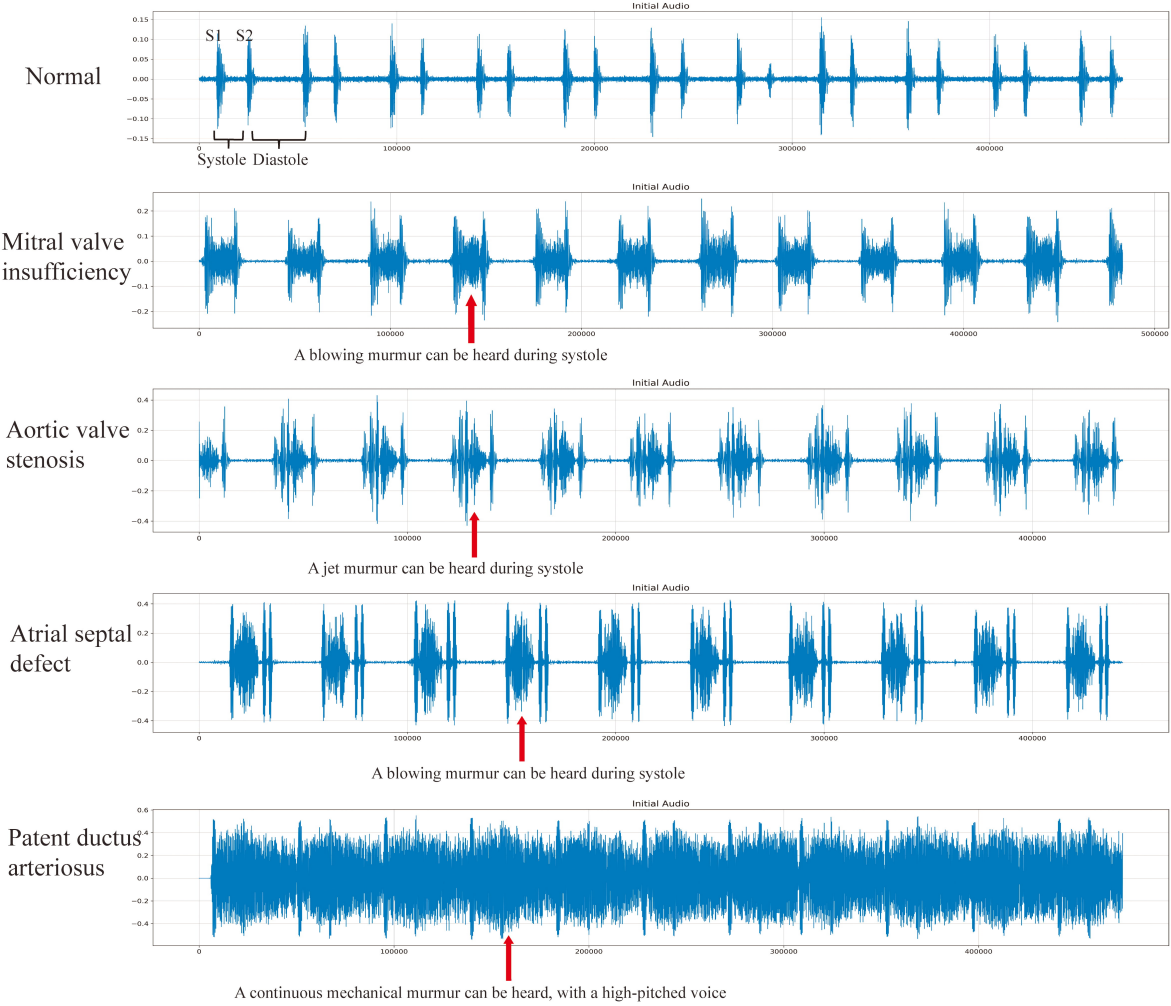
**Normal phonocardiogram and different heart disease phonocardiograms**. Note: The figure shows the phonocardiogram of a normal heart, some valvular heart disease and some congenital heart disease. The red arrow points to the murmur. S1: First Heart Sound; S2: Second Heart Sound.

Heart sounds are formed by vibrations caused by cardiovascular activities such as heart contractions, heart valve closures, and ventricular wall compressions. According to the order of occurrence in the cardiac cycle, heart sounds are divided into four components: the first heart sound (S1), the second heart sound (S2), the third heart sound (S3) and the fourth heart sound (S4) [8, 9]. In cardiac physiology, the period between S1 and S2 in the same cardiac cycle is referred to as systole, while the period between S2 and the S1 in the subsequent cycle is referred to as diastole. Heart murmur refers to the abnormal sound produced by the vibration of the ventricular wall, valves or blood vessels due to the turbulence of blood in the heart or blood vessels during systole or diastole, in addition to heart sounds, which are noises with different frequencies, different intensities and longer durations. Fig. 1 shows phonocardiograms (PCGs) of different diseases.

## 3. Principles of AI-Based Cardiac Auscultation

Cardiac auscultation is a qualitative method for assessing heart sounds, heart rate, pericardial friction sounds, etc. The advent of the digital stethoscope, augmented with analytical software, has revolutionized its utility, enabling objective and quantitative assessments of cardiac function in a clinical setting. Automated heart sound analysis in clinical applications usually consists of three steps: pre-processing, segmentation and classification. Pre-processing includes denoising, down-sampling, and normalizing data; Segmentation includes audio cutting and feature extraction; and classification model construction includes network building and model training.

### 3.1 Heart Sound Preprocessing

The audio data production standards of different datasets vary greatly, and the external interference produced by high-frequency and low-frequency environmental noises, human voices and heart sounds greatly restricts the heart sounds that can be collected by electronic stethoscopes during auscultation. The signal and noise generated after performing the wavelet transform not only change the scale of the wavelet coefficients but also decrease the accuracy of the cardiac signal analysis. This eventually leads to differences in the various audio signals, mainly in the audio sampling rate, number of channels, length, and self-contained noise reduction. Therefore, data preprocessing is needed for all audio files, and these differences need to be addressed before analysing the obtained datasets [10, 11].

The heart sound signal is first denoised to improve its signal-to-noise ratio; this is often executed with different filter thresholds and fixed thresholds for signal denoising. Next, the data need to be normalized. At present, the most commonly used normalization methods include Z score normalization, min-max normalization, and the functional transformation method. For unbalanced datasets, the original data classification imbalance is often addressed through undersampling, which aims to select a part of the data from the majority set and combine these data with the rest of the dataset to form a new dataset [12].

### 3.2 Heart Sound Segmentation

From the perspective of signal processing, a heart sound is a quasiperiodic nonsmooth random signal that consists of a mixture of normal heart sounds, murmurs and noise. The distinction between normal and abnormal heart sounds mainly lies in the identification of murmur features, so the extraction of effective murmur features from the collected heart sounds is critical for studying these sounds. In contrast to methods for addressing general pattern recognition problems, most heart sound analysis algorithms first segment murmurs before extracting heart sound features. Training a computer to think and solve problems like a human involves, to some extent, mimicking the thought process of the human brain. When interpreting heart sounds, human experts distinguish between S1 and S2 based on pitch, intensity and duration and finally identify the systole and diastole; this is similar to the process of computational analysis. In traditional signal processing methods, heart sound segmentation is performed by the Hilbert transform, hidden semi-Markov models (HSMMs), the average Shannon energy envelope algorithm, the Viola integration method, the short-time modified Hilbert transform algorithm, etc. [4, 13]. In recent years, several machine learning methods have been developed for heart sound segmentation. Algorithms based on logistic regression (LR) combined with hidden Markov models, genetic algorithms for spectral change detection, end-to-end methods based on convolutional long short-term memory (CLSTM) networks, and deep convolutional neural networks (CNNs) for U-Net segmentation have been established for heart sound segmentation [14, 15, 16, 17].

Traditional signal processing methods are efficient only if certain assumptions, such as finite-order linear system filtering, complex-domain Gaussian-distributed speech and noise, and band independence, are valid for the given application scenario and the statistics used in filtering can be accurately estimated. While machine learning methods do not always require these assumptions, the core of a machine learning model is a complex, nonlinear function; thus, these models can often achieve better results in real scenarios in which adequate training sets are available. However, machine learning models tend to perform less robustly without effective constraints and sufficient training data; moreover, systems that perform well in certain cases may perform poorly in other scenarios. In addition, the performance of machine learning methods is related to the utilized optimization metrics; for example, deep learning systems that use the signal-to-noise ratio as the main optimization metric may have large signal distortions that may be detrimental to heart sound segmentation. Therefore, methods based on a combination of traditional signal processing techniques and machine learning techniques can utilize the advantages of the underlying methods while addressing their limitations, allowing these approaches to perform heart sound segmentation in an accurate and efficient manner [18].

Feature extraction and feature selection can be used to accurately segment heart sounds and classify diseases. Theoretically, the classification performance should improve as more features are input during the training process. In practice, the classification performance decreases when the number of feature inputs exceeds a certain value after the number of training samples is set. Features are characteristics of the human brain that can be used to automatically identify and distinguish between objects, and they are similar in concept to variables in regression analysis. The features that can be recognized by machines are often in the form of numbers or symbols, while human experts extract physiological or pathological information from heart sounds through features such as the heart rate, heart rhythm, murmur timing and shape, heart sound frequency and the presence of additional heart sounds [19].

Alqudah *et al*. [20] demonstrated that higher-order spectral analysis methods in the field of digital signal processing extract significantly better features than lower-order feature extraction methods such as the short-time Fourier transform and wavelet transform. In addition, the second-order spectral method is the most widely used approach among the higher-order spectral methods, as it can effectively suppress phase relations in signals while detecting and quantifying the phase coupling of non-Gaussian signals. In recent studies, attention maps have been obtained by extracting features from data through self-attention mechanisms. This enables the derivation of the importance levels of different local information in the whole input image [21].

## 4. Classification Model Construction

**Fig. 2.**
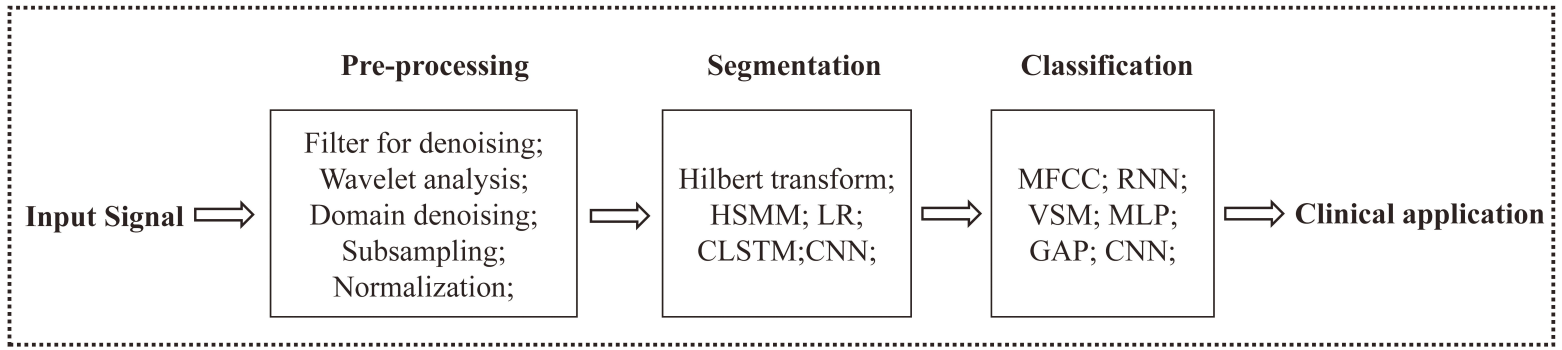
**Structural diagram of the heart sound identification process**. Note: HSMM, Hilbert transform, hidden semi-Markov model; CLSTM, convolutional long short-term; CNN, convolutional neural network; MFCC, Meier spectral coefficient; GAP, global average pooling; MLP, multilayer perceptron; LR, logistic regression; CNN, convolutional neural network; RNN, recurrent neural network; VSM, vector space model.

Traditional heart sound classification algorithms require that the feature extraction operators be manually set (Fig. 2), and such methods generally lack model generalizability and have limitations in terms of nonlinear data feature extraction. In recent years, scholars have proposed transforming the original heart sound signal into a two-dimensional heart sound time-frequency map with some transformations, such as the short-time Fourier transform, wavelet transform, and mel-frequency cepstral coefficients (MFCCs) [5, 22], and training deep convolutional networks in the frequency domain for classification purposes [23, 24].

Recently, the transformer and multilayer perceptron (MLP) techniques in deep learning have attracted the attention of many researchers. Traditional convolutional neural networks (CNNs) can automatically extract local information from data by conducting local weight sharing among the convolutional kernels; however, these models have difficulty accessing global information. Analyses of the second-order spectral feature maps of heart sound data have shown that in addition to certain local saliency characteristics exhibited by heart sound signals, global distribution characteristics are crucial for heart sound classification. An attempt has been made to design a hybrid model by using an MLP algorithm, global average pooling (GAP), and the convolutional technique instead of self-attention when constructing the training model [22]. The network was divided into three parts with different perceptual abilities, i.e., a global perceptron (GP), a partition perceptron (PP), and a local perceptron (LP), to classify the heart sound signals in all directions. Deploying deep learning algorithms to the cloud is a major trend in future research.

## 5. Heart Sound Datasets

The scarcity of heart sound data, particularly the unavailability of publicly accessible and high-quality heart sound databases, poses a significant challenge to the development and evaluation of AI auscultation algorithms, intelligent heart sound diagnosis and analysis technology, and auscultation screening applications [20]. A review of the commonly used heart sound databases that are available on the internet is presented in Table 1. Current heart sound databases have low applicability due to differences in electronic stethoscopes, timing, location, etc., and establishing a standardized heart sound database is the cornerstone of future intelligent heart sound research.

**Table 1.** **Detailed profiles of the utilized databases**.

Dataset	Sensor	Date of Publication	Number of Recordings	Age (year)	Rate (HZ)	Disease	Position	Website
Pascal Challenge	Digi Scope	2011	656	0–17	4000	CHD	Four typical positions	http://www.peterjbentley.com/heartchallenge/index.html
HSCT -11	ThinkLabs Rhythm	2016	206	-	11,025	Unknown	Four typical positions	http://www.diit.unict.it/hsct11/
Digiscope	Litmann 3200	2019	29	0–17	800–22,050	Unknown	Auscultatory mitral area	http://www.peterjbentley.com/heartchallenge/
HSS	EKO-CORE	-	170	-	4000	VHD	Four typical positions	http://www.compare.openaudio.eu/
CirCor	Litmann 3200	2021	1568	0–30	4000	CHD	Four typical positions	https://www.physionet.org/content/circor-heart-sound/1.0.1/
PhysioNet/CinC								https://www.physionet.org/content/challenge-2016/1.0.0/
AADHSDB	Littmann E4000	2015	151	-	4000	CHD	Tricuspid area	
MITHSDB	WAM E-stethoscope	2007	121	-	44,100	VHD	Nine different positions	
AUTHHSDB	AUDIOSCOPE	2014	45	18–90	4000	VHD	Apex	
TUTHSDB	Littmanns 3200	2013	44	-	4000	VHD	Four typical positions	
UHAHSDB	Prototype stethoscopes	2013	55	18–40	4000	VHD	Unknown	
DLUTHSDB	Littmann3200	2012	509	4–88	8000	CHD	Multiple positions at chest	
SUAHSDB	JABES	2015	112	16–88	800–22,050	Unknown	Apex	
SSHHSDB	Unknown	-	35	-	8000	VHD	2nd intercostal	
SUFHSDB	JABES	2015	225	23–35	4000–44,100	Foetal	Maternal abdomen	

Notes: HSCT -11: Heart Sounds Catania 2011 Database; HSS: Heart Sound Signal dataset; PhysioNet: Research Resource for Complex Physiologic Signals; CinC: Computing in Cardiology Challenge; PhysioNet/CinC Challenge Database: This database includes nine independent databases: the Aalborg University heart sounds database (AADHSDB), the Massachusetts Institute of Technology heart sounds database (MITHSDB), the Aristotle University of Thessaloniki heart sounds database (AUTHHSDB), the Khajeh Nasir Toosi University of Technology heart sounds database (TUTHSDB), the University of Haute Alsace heart sounds database (UHAHSDB), the Dalian University of Technology heart sounds database (DLUTHSDB), the Shiraz University adult heart sounds database (SUAHSDB), the Skejby Sygehus Hospital heart sounds database (SSHHSDB), and the Shiraz University foetal heart sounds database (SUFHSDB). CHD, congenital heart disease; VHD, valvular heart disease.

## 6. Electronic Stethoscope

An electronic stethoscope is an important tool for achieving AI-based heart sound diagnosis. The stethoscope is an instrument that is commonly used by all doctors. While its invention dates as far back as 1816, several technological evolutions have occurred over the past 200 years, the latest of which is the electronic stethoscope, which was first developed in the early 1990s [25]. Since then, electronic stethoscopes with various functions have been proposed. Table 2 shows the advantages and disadvantages of different stethoscopes in terms of their recording frequencies, communication techniques, data losses, filtering techniques and environmental filtering techniques [26, 27, 28, 29].

**Table 2.** **Characteristics of different electronic stethoscopes in terms of various aspects**.

Electronic stethoscope	Recording frequencies	Communication technology	Data loss	Filtering techniques	Advantages and disadvantages
3M Littmann 3200	Bell mode (20–1000 Hz)	Bluetooth	Acceptable	ANC friction noise dampening	The stored records may be accessed by outside users by exporting them to WAVE audio files.
	Diaphragm mode (20–2000 Hz)				If the LED display is damaged, it is extremely difficult to record and adjust settings.
	Extended mode (20–2000 Hz)				
ThinkLabs One Digital	20–2000 Hz	Audio interface	Excellent	Manipulable filtering range (piezoelectric sensors)	Amplifies sounds by 100 times; small, with an easily portable design; phones can be used to record sounds.
					Cannot record sounds by itself; requires another device such as a smartphone or an iPad to record sounds; noise reduction does not reach expectations.
Jabes	Bell mode (20∼200 Hz)	Audio interface	Acceptable	Unknown	Reasonable price.
	Diaphragm mode (200∼500 Hz)				Complex operation.
	Wide mode (20∼1000 Hz)				
Eko Core	20–2000 Hz	Bluetooth	Excellent	ANC friction noise dampening	Easily identifies heart murmurs with Eko’s automated detection software.
					Cell phone support required to work; cannot be used independently.
Welch Allyn Elite	20–2000 Hz	Bluetooth	Excellent	Piezoelectric sensors	Comfortable to wear; excellent noise reduction ability.
					Not commercially available; complex operation; possesses a single function.
HD Steth	Bell mode (50–200 Hz)	Bluetooth	Unknown	Unknown	Performs concurrent auscultation and ECG; employs AI to produce superior visualization results.
	Dia mode (50–600 Hz)				Complex operation.
	Lung mode (20–2000 Hz)				Expensive.

Note: Advantages and disadvantages of different stethoscopes in terms of their recording frequencies, communication techniques, data losses, filtering techniques and environmental filtering techniques. ANC, active noise cancellation; ECG, electrocardiogram; LED, light emitting diode; and HZ, hertz.

## 7. Applications in CHD Diagnosis

The high incidence of CHD and the dangers leading to poor prognosis are widely recognized. For young children, early detection and treatment are important to reduce the mortality rate of CHD. At present, the CHD diagnosis process is divided into two steps: first, for the initial diagnosis, the doctor makes a preliminary judgement on whether the patient has CHD through cardiac auscultation; then, the initial diagnosis is confirmed by echocardiography in suspected cases. Most patients with CHD are not diagnosed in the early stages of life due to the lack of specific symptoms [30]. This prevents the infant from receiving timely and effective surgical repair or palliative care. Although echocardiography is the gold standard for verifying CHD cases, it usually takes more than 10 minutes to complete [30]. Therefore, in resource-limited areas, it is impractical to perform an echocardiogram on every screening subject. In many areas, community screening for congenital heart disease is often performed by auscultation [31]. With the development of imaging technology, an increasing number of physicians are losing the skill of auscultation [32], and in the absence of symptoms of congenital heart disease, physicians will not perform ultrasound and other tests on patients. In addition to these reasons, the unequal distribution of medical resources is also an important one, thus creating a paradox: the number of young children who need to be screened is large, but few doctors have the required clinical auscultation experience [33]. Some AI-based auscultation applications for CHD diagnosis are summarized in Table 3 (Ref. [4, 5, 21, 22, 23, 24, 34, 35, 36, 37, 38, 39, 40]).

**Table 3.** **Intelligent auscultation methods for diagnosing CHD**.

Author	Algorithm	Controls (cardiac patients)	Cardiac pathology	ACC
Wang *et al*. [4]	ANN	86 (62)	An intelligent method for diagnosing paediatric CHD murmurs was developed.	93%
SUN *et al*. [34]	MFCC–HMM	227 (60)	A simple and efficient diagnostic system was proposed for diagnosing VSDs.	92.1–99.0%
Gharehbaghi *et al*. [22]	STGNN	50 (22)	An automated screening tool for identifying children with isolated bicuspid aortic valves (BAVs) was developed.	87.4%
Lai *et al*. [35]	Unknown	106	The confirmation of the novel computational algorithm’s high quality and real-world robustness for the assessment of paediatric murmurs was established.	87%
Aziz *et al*. [5]	SVM	280 (140)	The proposed methodology achieved high accuracy in terms of classifying patients with ASDs, patients with VSDs, and normal subjects.	95.4%
Gómez *et al*. [36]	XGBoost	265 (128)	This study investigated the feasibility of using artificial intelligence (AI) for detecting patent ductus arteriosus (PDA) based on neonatal phonocardiogram (PCG) data.	78%
Son *et al*. [23]	SVM, DNN, KNN	1000 (800)	Heart sound signals were classified using multiple features.	97.9%
Zhu *et al*. [24]	MFCC, LPCC	140 (69)	The features extracted by using the MFCC method were better than those obtained by using the LPCC approach.	93.02%
Patidar *et al*. [21]	LS-SVM	326 (163)	Cardiac sound signals were characterized to diagnose septal defects based on a novel feature set.	99.35%
Chourasia *et al*. [37]	Unknown	25	CHD was identified based on foetal phonocardiography (fPCG) signals.	88%
Ahmad *et al*. [38]	SVM, KNN	283 (108)	Heart murmurs were detected and the associated cardiovascular disorders were classified based on heart sound signals.	92.6%
Babaei *et al*. [39]	ANN	372 (270)	Two effective classification strategies have been proposed for the discrimination of heart valve abnormalities. The first approach involves the use of neural network training, while the second method employs statistical averaging on an efficiently decomposed version of clinical samples.	94.42%
Lv *et al*. [40]	CNN	1362 (1149)	A high accuracy rate for the detection of abnormal heart sounds was achieved using the AI-AA platform, which enables remote and automatic auscultations. The results also demonstrated excellent agreement with expert auscultations.	96%

Notes: SVSD, a small VSD; MVSD, a moderate VSD; LVSD, a large VSD; NM, normal; ANN, approximate nearest neighbours; MFCC–HMM, Mel-frequency cepstral coefficient-hidden Markov model; STGNN, spatial-temporal graph neural network; SVM, support vector machine; XGBoost, extreme gradient boosting; DNN, dynamic neural network; KNN, K-nearest neighbours; LPCC, linear prediction cepstral coefficient; LS-SVM, least-squares support vector machine; CNN, convolutional neural network; CHD, congenital heart disease; VSD, ventricular septal defect; BAV, bicuspid aortic valve; ACC, accuracy.

### 7.1 Applications in Prenatal Diagnosis

Neonatal screening is crucial for obstetrics, and prenatal screening is often performed clinically by complex methods. The debate over which images of obstetric ultrasound should be included in the “routine” examination of the foetal heart affects the sensitivity of such examinations [41, 42] and detection rates remain low [43]. In addition, certain lesions, such as transposition of the great arteries (TGA), can be difficult to detect for physicians without expertise in CHD [44]. Mellander *et al*. [45] showed that in a population of infants requiring cardiac catheterization or surgery within the first 2 months of life (excluding patients diagnosed prenatally), 57% of infants with CHD had been discharged home at 72–120 hours of life. Combining the above reasons, any method that helps improve the screening reliability is worth investigating. According to a recent systematic review of published literature encompassing data from eight centres and 36,237 pregnancies, it was found that the overall rate of detection of major congenital anomalies at 11–13 weeks was 29% for cases involving more than 1000 pregnancies. The pooled cardiac defect detection rate was 17% [46]. Early CHD identification approaches with heart sound signal processing methods have been reported [47, 48]. Kovács *et al*. [49] researched prenatal heart sounds to diagnose foetal heart disease, and in 2015, they proposed a remote diagnosis method for foetal congenital heart disease with the help of auscultation. Although the sample sizes in the relevant studies are small, the diagnosis of murmurs in the foetal life stage with intelligent auscultation methods is a challenging task. Early CHD screening based on foetal heart sound data has been studied, but these studies are still scarce, and more research is needed. These studies are limited to the diagnosis of CHD, and there is still a gap in the field in terms of prognosis and severity assessment of CHD.

### 7.2 Screening for CHD in a Population

When screening a population, traditional methods of cardiac auscultation alone are often not accurate enough. According to the literature, the sensitivity and specificity of auscultation screening for congenital heart disease are 75.0% and 99.0%, respectively [30]. Lillian S.W. Lai *et al*. [35] collected heart sound data from 106 patients with CHD and healthy patients and obtained phonocardiograms for each case, and used this data to train an intelligent model, the model achieved a sensitivity of 87%, a specificity of 100%, a positive predictive value of 100%, a negative predictive value of 90%, and an overall accuracy of 94%.

However, smart stethoscopes that discriminate only between normal and abnormal sounds have limited clinical applicability. Shuping Sun and colleagues aimed to diagnose small, medium, and large VSDs using classification boundary curves and an elliptical model based on heart sound feature extraction. The elliptical model classified normal patients and patients with small, medium, and large VSDs better than the other five tested models (accuracies of 99%, 95.5%, 92.1%, and 96.2%, respectively). There are nuances in the auscultation of heart murmurs in CHD, but it is clear that AI approaches can obtain improved diagnostic accuracy for physicians at all experience levels [34].

## 8. Applications in VHD Diagnosis

VHD is usually a slowly progressive, chronic disease that may be asymptomatic in its initial stages. The collected data have repeatedly shown that most patients are diagnosed with advanced-stage disease when they are symptomatic or have complications (e.g., ejection dysfunction). Several factors may lead to the delayed diagnosis of VHD, including patients’ inadequate knowledge of the condition and clinicians’ underutilization of cardiac auscultation. Even with experienced clinicians, the sensitivity (up to 43%) and specificity (69%) of physician auscultation for the diagnosis of significant VHD are inadequate [6]. Digital stethoscopes improve murmur detection by converting sounds into electronic signals that can be further amplified, filtered and digitized [50, 51] (Table 4, Ref. [12, 13, 22, 31, 35, 52, 53, 54, 55, 56, 57]).

**Table 4.** **Intelligent auscultation in diagnosing VHD**.

Author	Algorithm	Controls (cardiac patients)	Cardiac pathology	ACC
Thompson *et al*. [52]	Unknown	603 (374)	A quantitative and objective evaluation of a heart murmur detection algorithm was conducted using virtual clinical trials.	88%
Lai *et al*. [35]	Unknown	106 (81)	This study evaluated the efficacy of a new algorithm designed to automatically classify murmurs detected in phonocardiograms (PCGs) acquired from paediatric populations.	94%
Sengur *et al*. [53]	PCA, AIS, KNN	215 (120)	A medical decision support system with normal and abnormal classes was developed.	95.9%
Asmare *et al*. [54]	RBF, SVM	251 (124)	A machine learning-based automated screening approach for rheumatic heart disease (RHD) was developed, which enables non-medically trained individuals to use it outside clinical settings.	96.2%
Maragoudakis *et al*. [13]	RF	198 (160)	A new ensemble classification approach was proposed, integrating random forests with the Markov blanket model, for the automated diagnosis of aortic and mitral heart valve diseases, based on low-cost and easily obtainable heart sound signals.	98.67%
Chorba *et al*. [31]	ResNet24	962 (141)	This study aimed to evaluate the efficacy of a deep learning algorithm in detecting heart murmurs and clinically significant valvular heart diseases (VHDs) using recordings obtained with a commercially available digital stethoscope platform.	AS: 95.2%, MR: 86.5%
Singh *et al*. [12]	CNN	631 (214)	A comprehensive approach comprising a cost-effective digital stethoscope, mobile application and cloud-based software for audio processing, training and classification was formulated to detect valvular heart disorders at an early stage. This approach has the potential to be expanded to other ailments that depend on auscultation as a diagnostic technique, including respiratory disorders.	95%
Comak *et al*. [55]	SVM, ANN	215 (120)	A decision support system that aids physicians in evaluating aortic and mitral heart valve disorders was developed.	94.37%
Gharehbaghi *et al*. [22]	Unknown	45 (15)	This study presented a processing method for discriminating between murmurs caused by AS and PS.	93.3%
Maglogiannis *et al*. [56]	SVM	198 (84)	A diagnostic system that utilizes support vector machine (SVM) classification of heart sounds to identify heart valve diseases was proposed. This system is capable of performing a challenging diagnostic task that is significantly more complex than merely identifying the presence of a heart valve disease.	91.43%
Voigt *et al*. [57]	CNN	200 (100)	A deep learning-based auscultation approach was developed that predicted significant AS with similar accuracy to that of cardiologists.	95%

Notes: PCA, principal component analysis; AIS, automatic identification system; RBF, radial basis function; RF, random forest; ResNet, residual network; ANN, approximate nearest neighbours; CNN, convolutional neural network; KNN, K-nearest neighbours; SVM, support vector machine; RHD, rheumatic heart disease; AS, aortic stenosis; PS, pulmonary stenosis; MR, mitral regurgitation; ACC, accuracy.

Thompson *et al*. [52] selected 3180 heart sound recordings from 603 clinic visits from the Johns Hopkins Cardiac Auscultatory Recording Database. The detection sensitivity and specificity of patients with pathological murmurs were 93% and 81%, respectively, with an accuracy of 88%. However, data that were considered “noisy” or lacking audible murmurs were removed prior to testing, which artificially improved the performance of the algorithm compared to that achieved in real-world settings [52]. In addition to diagnosing valvular disease, computer-assisted auscultation appears to be a relevant support tool for detecting pathological murmurs and appropriately referring patients for further evaluation (93% referral sensitivity and 79% specificity) according to Watrous *et al*. [58]. The performance varied according to the deterministic measurements of the algorithm, patient ages, heart rates, murmur intensities, and chest recording locations [52]. In a separate investigation [59], Gharehbaghi *et al*. [22] employed a combination of two deep learning methods, static and dynamic time-varying neural networks, to analyse phonocardiogram (PCG) data. The model was applied to evaluate 140 children with congenital heart disease (CHD) and 50 elderly patients with aortic stenosis (AS), achieving an accuracy of 84.2% and a sensitivity of 82.8%.

Although AI-based cardiac auscultation can help in the diagnosis of VHD, obesity and diseases that affect auscultation (e.g., chronic lung disease) may affect the quality of the obtained sound, leading to inaccurate results. However, because screening large populations is much less expensive than using echocardiography data, this screening process reduces the need for trained health professionals and does not require specialized health care facilities.

## 9. Limitations

Some limitations still need to be addressed before the technology may be used more widely: First, algorithms are often sensitive to the type of stethoscope used and the quality and range of data obtained, and the same algorithm often produces different results for the interpretation of signals obtained from different stethoscopes [20]. In addition, AI-based stethoscopy algorithms should be conducted in collaboration between researchers and medical experts to avoid research compartmentalization [60]. Most importantly, intelligent auscultation should ultimately be used for clinical purposes, yet most of the existing studies have focused on theoretical algorithms rather than practical applications [32]. Another important issue is the lack of a common, authoritative and comprehensive database to compare algorithms and address data imbalances, as each study is relatively independent and there are few systematic and objective evaluations of acquisition environments, parameters and methods [61, 62]. The field will also involve the concept of ethics, as the black-box nature of AI methods leads to unexplainable algorithms without sufficient theory to support their widespread use in clinical settings [63], and one of the biggest challenges is the decreasing frequency of stethoscope use in actual clinical practice, many imaging tests having long since replaced acoustically driven stethoscopes [32].

## 10. Future Perspectives

In terms of recent research, AI-based methods have rarely been applied in clinical settings, and because AI lacks the human-like ability to think about and explore different diseases, AI-based approaches cannot yet replace clinicians and independently complete treatments. The following are future research directions in this field: PCG data can be applied when differentiating between innocent and pathological murmurs is difficult. In such cases, the use of PCGs may increase or decrease the level of suspicion and prompt further investigation or reassurance [19]. In settings with limited access to diagnostic tools, PCG signals may be used to confirm the clinical presentation of VHD for referral to centres with more advanced diagnostic capabilities, rather than for screening purposes [27]. The establishment of high-quality heart sound databases for multiple cardiac diseases and the creation of uniform standards for this purpose are important directions for the future development of this field [52]. Crucially, establishing a unified heart sound processing scheme and solving the problem of interpretability of intelligent models is one of the biggest problems in translating intelligent auscultation into clinical applications.

## 11. Conclusions

Smart VHD and CHD auscultation has been used initially in some studies with good results, but currently faces some problems that need to be solved by conducting more studies in the future.

## Abbreviations

AI, artificial intelligence; ASD, atrial septal defect; VSD, ventricular septal defect; PDA, patent ductus arteriosus; VHD, valvular heart disease; CHD, congenital heart disease; PCG, phonocardiogram; HSMM, Hilbert transform, hidden semi-Markov model; CLSTM, convolutional long short-term; CNN, convolutional neural network; EEG, electroencephalogram; ECG, electrocardiogram; EMG, electromyography; MFCC, Meier spectral coefficient; GAP, global average pooling; GP, global perceptron; PP, partition perceptron; LP, local perceptron; ANC, active noise cancellation; LED, light emitting diode; HZ, hertz; HMM, Mel-frequency cepstral coefficients-hidden Markov model; STGNN, spatial-temporal graph neural network; SVM, support vector machine; XGBoost, extreme gradient boosting; DNN, dynamic neural network; KNN, K-nearest neighbours; LPCC, linear prediction cepstral coefficient; LS-SVM, least-squares support vector machine; CNN, convolutional neural networks; ACC, accuracy; ROC, receiver operating characteristic; AS, aortic stenosis; PS, pulmonary stenosis; MR, mitral regurgitation.
